# Dacarbazine and the Agonistic TRAIL Receptor-2 Antibody Lexatumumab Induce Synergistic Anticancer Effects in Melanoma

**DOI:** 10.1371/journal.pone.0045492

**Published:** 2012-09-20

**Authors:** Birgit Engesæter, Olav Engebraaten, Vivi Ann Flørenes, Gunhild Mari Mælandsmo

**Affiliations:** 1 Department of Tumor Biology, Oslo University Hospital, Oslo, Norway; 2 Department of Oncology, Oslo University Hospital, Oslo, Norway; 3 Institute for Clinical Research, Medical Faculty, University of Oslo, Oslo, Norway; 4 Department of Pathology, Oslo University Hospital, Oslo, Norway; 5 Department of Pharmacy, Faculty of Health Sciences, University of Tromsø, Tromsø, Norway; Bauer Research Foundation, United States of America

## Abstract

Mapatumumab and lexatumumab (targeting death receptor 4 (DR4) and 5 (DR5), respectively) are agonistic TRAIL receptor antibodies that induce apoptosis in a wide range of cancer cells. The potency of mapatumumab and lexatumumab was assessed in mono therapy protocols, and the ability to sensitize for dacarbazine (DTIC) treatment was explored in ten different melanoma cell lines. Our data indicated that melanoma cell lines tend to be resistant to mapatumumab, most likely due to low expression of DR4, while a dose dependent response to lexatumumab was observed. Combining DTIC and lexatumumab induced an additive or synergistic effect on cell death in the various melanoma cell lines. The synergistic effect observed in the FEMX-1 cell line was related to enhanced cleavage of Bid in parallel with elevated expression of the pro-apoptotic proteins Bim, Bax and Bak. Furthermore, the anti-apoptotic proteins Bcl-XL, cIAP-1, XIAP and livin were down regulated. Cleavage of Bid and down regulation of cIAP-2 and livin were observed *in vivo*. Altogether, these data suggest a change in the balance between pro- and anti-apoptotic proteins favoring induction of apoptosis. In the more therapy resistant cell line, HHMS, no changes in the pro- and anti-apoptotic proteins were observed. FEMX-1 xenografts treated with DTIC and lexatumumab showed reduced growth and increased level of apoptosis compared to the control groups, providing arguments for further evaluation of this combination in melanoma patients.

## Introduction

The incidence of melanoma has increased over the past three decades, and the death rate continues to rise faster than for most other cancer types. The prognosis of malignant melanoma is strongly related to the stage at which it is detected. The overall survival is excellent for primary cutaneous melanoma patients diagnosed early and treated by adequate surgery, while prognosis of disseminated melanoma is poor, with five year survival below 6% and median survival less than one year [1]. Dacarbazine (DTIC) is the first-line therapy for metastatic melanoma [2], but for most patients (80–87%) DTIC treatment fails. Thus, new treatment strategies for this patient group are needed. Two therapeutic regimens, vemurafenib (targeting the B-RAF V600E mutation) and combination therapy utilizing ipilimumab (antibody directed against cytotoxic T lymphocyte-associated antigen 4 (CTLA-4) and DTIC, have resulted in an improved overall survival compared to DTIC mono therapy [3], [4], [5]. However, the above mentioned regimens are not suitable for the whole patient group due to toxicity, lack of the V600E mutation and development of resistance, and other treatment strategies are therefore still required.

Conventional radio- and chemotherapy aim to eliminate the tumor cells through apoptotic cell death. Many melanoma cells have, however, accumulated multiple mutations reducing their ability to induce apoptosis. Pro-apoptotic proteins have been reported to be either down-regulated, as for instance initiator caspases, death receptor 4 (DR4) and BH3-only molecules (e.g. Bad, Bim, Puma and Noxa) [6], [7], [8], or inactivated, as p53 [9], while anti-apoptotic proteins, such as Mcl-1, Bcl-XL and various Inhibitor of Apoptosis Proteins (IAPs) are reported to be over-expressed [10], [11], [12], [13]. TRAIL (TNF-related apoptosis inducing ligand) is a promising cancer drug candidate showing high therapeutic index due to strong activity in cancer cells while normal cells usually are spared [14]. TRAIL binds to death receptor 4 and 5 (DR4 and DR5) leading to activation of caspase-8 and subsequently caspase-3. Caspase-3 can either be activated directly by caspase-8 (Type I pathway) or through activation of the intrinsic pathway depending on mitochondrial dysfunction, and referred to as the Type II death receptor pathway of apoptosis [15]. There are several reports demonstrating tumor growth suppression *in vivo* when applying TRAIL as mono therapy or in combination with other drugs, and recombinant TRAIL or corresponding agonistic antibodies are in clinical evaluation for various cancer types [14].

In the present study the potency of agonistic TRAIL receptor antibodies was assessed and the antibodies effect on DTIC sensitivity was explored. By combining DTIC and agonistic TRAIL receptor antibodies, we demonstrated increased cell death compared to the mono treatments. *In vitro* down regulation of XIAP, livin and cIAP-1, in parallel with up regulation of Bim, tBid, Bak and Bax, may explain the increased sensitivity, while minimal effect on the pro- and anti-apoptotic molecules were observed in more therapy resistant cells. *In vivo* the combination resulted in significant reduced tumor growth. Increased cleavage of Bid in addition to reduced expression of livin and cIAP-2 may explain the enhanced caspase activation and the reduced growth of the xenografts. The obtained results are promising and suggest that the combination of DTIC and lexatumumab should be subjected for further preclinical testing and possibly considered for translation into clinical evaluation.

## Materials and Methods

### Reagents

DTIC supplied by Medac (Hamburg, Germany) was dissolved in sterile water. IgG isotype control, mapatumumab and lexatumumab (formerly HGS-ETR1 and HGS-ETR2, respectively) were provided from Human Genome Sciences, Rockville, MD. IgG isotype control used in the animal studies was supplied by Sigma Chemical Company (St.Louis, MO, USA).

### Cell Lines and Culture Conditions

The cell lines HHMS, RMS, FEMX-1 and LOX were established from metastatic lesions of malignant melanoma patients treated at the Norwegian Radium Hospital [16], [17]. The WM35, WM115, WM239 and WM1341 cell lines were kindly provided by Dr. Meenhard Herlyn (Wistar Institute, Philadelphia, PA, USA, [18]), while A375 and SKMEL-28 were obtained from the American Type Culture Collection (Rockville, MD, USA). The normal human fibroblast cells, HuFib, were established by L- Bruckner_Tudeman (University of Münster, Germany). All cell lines were maintained in RPMI 1640 medium (Bio Whittaker), except for HuFib, which was cultivated in Dulbecco’s modified Eagle medium (DMEM. Bio Whittaker). Both media were supplemented with 10% fetal bovine serum (FBS, PAA Laboratories, Linz, Austria) and 2 mM L-glutamine (GibcoBRL, Paisley, UK). The cells were maintained at 37°C in a humidified atmosphere containing 5% CO_2_, and were routinely tested for mycoplasma infection.

### Agonistic TRAIL Receptor Antibodies and DTIC Exposure

Indicated melanoma cell lines were seeded in specified well-format depending on designated analysis the day before treatment. Various concentrations of the antibodies (0.01, 0.1, 1.0 or 10.0 µg/ml) or DTIC (10, 50 or 100 µg/ml) alone or in combination were added and the samples were analyzed or harvested at different time points depending on the subsequent analysis.

### Cell Viability

The growth inhibitory effect of the TRAIL receptor antibodies alone or combined with DTIC was measured by the use of CellTiter 96 Aqueous One solution (MTS assay, Promega, Madison, WI, USA). Cells were seeded in 96-well plates and treated as described in previous section. Seventy two hours after treatment CellTiter 96 Aqueous One Solution was added to the wells and the absorbance was measured at 490 nm after approximately 2 hours using a micro plate reader (Victor^2^ 1420 Multilabel Counter, Perkin Elmer). Viability of treated cells is reported as the percentage of viable cells relative to untreated control cells. Experiments were performed in four parallels and repeated at least in three independent biological experiments for each treatment condition.

### Calcusyn Analysis

We evaluated possible synergism using the Chou and Talalay combination index (CI) analysis, a well-established index to determine the interaction of two drugs. Non-exclusive treatments are defined as treatments affecting different targets or different sites of the same target. The CI value of nonexclusive treatments is calculated by the formula:

CI  =  (Da + Db)/(Dxa + Dxb) + Da*Db/Dxa*Dxb.

Da and Db are doses needed of treatment A and B to inhibit x% of cell proliferation as single treatments, and Dxa and Dxb are the doses of A and B to inhibit x% of cell growth in a combination regimen. Synergism is defined as more than the expected additive effect with CI<1 and antagonisms is defined as CI>1. CI values were analyzed using the Calcusyn software (BioSoft, Feruson, MO, USA). The inputs are the doses of the single treatments, the combination doses at different ratios, and the fractional inhibition (fraction affected (Fa)) of single and combination treatment. Fa  = (OD_490 control_ − OD_490 treated_)/OD_490 control_. Fraction of unaffected cells Fu = 1 − Fa.

### Clonogenic Assay

Clonogenic efficiency after treatment with lexatumumab and DTIC alone or in combination was determined. Cells were seeded in 6-well plates and treated as described above. Cells were grown for 2 weeks before they were washed in PBS and fixed in ice-cold methanol prior to staining with 0.04% crystal violet, washed in tap water and then air dried at room temperature. Colonies of ≥50 cells were counted to determine the surviving fraction using the following formulae: Plating efficiency, PE  =  number of clones counted in controls/number of cells plated. Surviving fraction, SF  =  number of clones in treated well/(number of cells seeded×PE). At least three parallels were scored in three independent biological experiments for each treatment condition. The surviving fractions are given as the mean ± standard error of the mean. The chosen plating density aimed to produce 20–100 surviving colonies in each well.

### Western Immuno-blot Analysis

Cells exposed to indicated treatment were lysed in lysis buffer (20 mM Tris-HCl pH7.5, 137 mM NaCl, 100 mM NaF, 10% Glycerol and 1% NP-40) supplemented with 2 mg/ml pepstatin, aprotinin (Sigma Chemical Company) and leupeptin (Roche Diagnostics, Mannheim, Germany) for 1 hour on ice before sonication. The debris was removed by centrifugation and the lysates stored at −70°C. Total cellular protein (15–50 µg) of each sample was separated in a NuPAGE®Novex® Midi Gel Bis-Tris 4–12% (Invitrogen, Carlsbad, CA, USA) and subsequently transferred to a nitrocellulose membrane using a iBlot® Dry Blotting System (Invitrogen, Carlsbad, CA, USA). The membranes were probed at 4°C overnight with primary *antibodies against:* cIAP-1 (*AF8181),* cIAP-2 (AF8171), XIAP (AF8221), survivin *(AF886),* livin (AF1161) (R&D system), Bad (#9292), Bak (#3814), Bax (#2774), Bcl-XL (#2762), Bid (#2002), Bik (#4592), Bim (#2819), caspase-3 (#9662), cleaved caspase-3 (#9664), caspase-8 (#9746), caspase-9 (#9502), Mcl-1 (#4572), PARP (#9542) (Cell Signaling Technology, Danvers, MA, USA) and Bcl-w (ab13525) (Abcam, Cambridge, UK). Appropriate horseradish peroxidase-conjugated secondary antibodies and SuperSignal West Dura Extended Duration Substrate (Thermo Scientific, Rockford, IL, USA) were used for visualization. Cell lysates from three independent biological experiments were prepared.

### Caspase Activity Assay

The activity of caspase 3/7 in FEMX-1 and HHMS cells was measured using Caspase-Glo 3/7 assay from Promega. Five thousand cells were seeded in 96-well optical bottom plates with white upper structure (Nunc, Roskilde, Denmark) and treated as described above. The activity was measured after 48 hrs in accordance with the manufacturer’s instructions. Assays were performed in triplicate and repeated at least three times.

### Ethics Statement

All procedures and experiments involving animals were approved by the National Animal Research Authority and were conducted according to the regulations of European Laboratory Animals Science Association. Nude female mice (BALB/c (nu/nu)) were bred inour rodent facility. The animals were kept in a specific pathogen-free environment, in positive pressure rooms with filtered and humidified air. The animals were kept under standard conditions, and food and water were supplied *ad libitum*.

### *In vivo* Studies

FEMX-1 xenografts were treated with DTIC 62.5 or 125 mg/kg (i.p.) once a week, IgG isotype control or lexatumumab 10 mg/kg (i.v.) twice a week or the combination of DTIC and lexatumumab or DTIC and IgG isotype control. The tumour size was measured twice a week by a calliper and the volume V was calculated as follows: V = W^2^×L×0.5, where W and L is tumour width and length, respectively. The mean tumour volume at the experimental initiation was 84.1 mm^3^. At the end of the experiments the mice were euthanized by dislocation of the neck.

### Immunohistochemistry

Three-µm sections made from formalin-fixed paraffin embedded tissues were immunostained using the Dako EnVision^TM^Flex+ System (K8012, Dako A/S, Glostrup, Denmark). Deparaffinization, rehydration and target retrieval were performed in one operation in a Dako PT-link and EnVision™ Flex target retrieval solution with high pH. To block endogenous peroxidase the sections were treated with Dako EnVision Peroxidase Block for 5 minutes. Sections were incubated for 30 minutes with monoclonal cleaved caspase-3 antibody (#9664, Cell Signaling, Beverly, MA) diluted 1∶100 or monoclonal PARP cleaved p85 fragment antibody, diluted 1∶100 (#g7341, Promega, Madison, WI). Thereafter, the sections were incubated with Dako EnVision™ FLEX+ mouse linker for 15 minutes followed by incubation with Dako EnVision™ FLEX/HRP for an additional 30 minutes. For visualization of staining, the sections were treated with 3′3-diaminobenzidine tetrahydrochloride (DAB) Chromogen (Dako), counterstained with haematoxylin, dehydrated and mounted from xylol with Richard-Allan Scientific Cyto seal XYL (Thermo scientific, MA, USA). Sections from a B cell lymphoma with known expression of cleaved caspase-3 was used as control positive control whereas WM35 human melanoma cells treated with 500 ng/ml Tricostatin A was used as positive control for cleaved PARP. Four semiquantitative classes were used to describe the number of stained tumor cells: absent, 0; <10%, 1; 10–50%, 2; >50%, 3. Intensity was evaluated as weak (1), moderate (2) and strong (3).

### Statistical Analysis

Mann-Whitney non-parametric test for independent samples were performed to statistically test differences in clonogenicity after DTIC or lexatumumab mono treatment relative to the combination treatment. For statistical analysis of the *in vivo* data the mean volume of the tumors was used for mice having two tumors. To test the statistical difference in tumor growth between the different groups repeated measures of variance with Dunnet’s test for adjustment for multiple comparisons was performed. Considering the low test power due to the low number of samples, we found it interesting to increase the test power by combining control groups. The same statistical analysis was performed between merged control groups (group 1: untreated tumors and lexatumumab treated tumors, group 2: DTIC-treated and IgG- and DTIC-treated tumors) and the group treated with lexatumumab and DTIC. Unequal variance was assumed. Results were considered statistically significant if p≤0.05. All statistical analyses were performed using SPSS 16.0 (Chicago, IL, USA).

## Results

### Melanoma Cell Lines Show Various Responses to Mapatumumab and Lexatumumab

**Figure 1:**
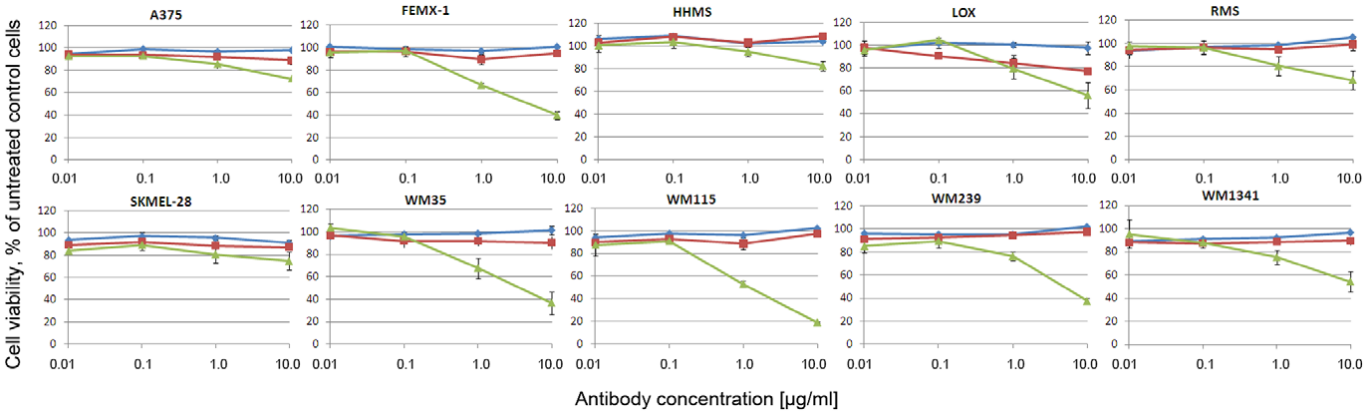
Sensitivity to agonistic TRAIL receptor antibodies in ten melanoma cell lines. The melanoma cell lines were exposed to increasing concentrations of isotype control antibody (diamonds), mapatumumab (squares) and lexatumumab (triangles) at 0.01, 0.1, 1.0 and 10.0 µg/ml and the cell viability was measured after 72 h. The percentage of living cells is expressed relative to untreated control cells. Four parallels were included in each assay, and at least three separate biological experiments were performed. The error bars represent mean ± SEM.

Ten melanoma cell lines were exposed to increasing concentrations of two different agonistic TRAIL-receptor antibodies, mapatumumab and lexatumumab, targeting DR4 and DR5 respectively. The cell viability was measured after 72 h by MTS assay (Figure 1), and indicated a general resistance to mapatumumab at all concentrations in all cell lines apart from LOX, while lexatumumab induced various degrees of cell death in the different cell lines. WM115 was the most sensitive to lexatumumab (80% reduction in cell viability at the highest concentration), while the growth of HHMS was only modestly affected. The expression of the death receptors DR4 and DR5 were analyzed in five selected cell lines (Table S1). With the exception of LOX cells, the cell lines had no, or very low, expression of DR4. Thus, the mapatumumab resistance observed in most of the cells could be related to the lack of DR4 expression. In line with this observation, mapatumumab affected the viability of the LOX cells slightly, inducing approximately 20% cell death. All cell lines studied expressed significant levels of DR5.

### Combined Treatment with Lexatumumab and DTIC Increases Cell Death

**Figure 2:**
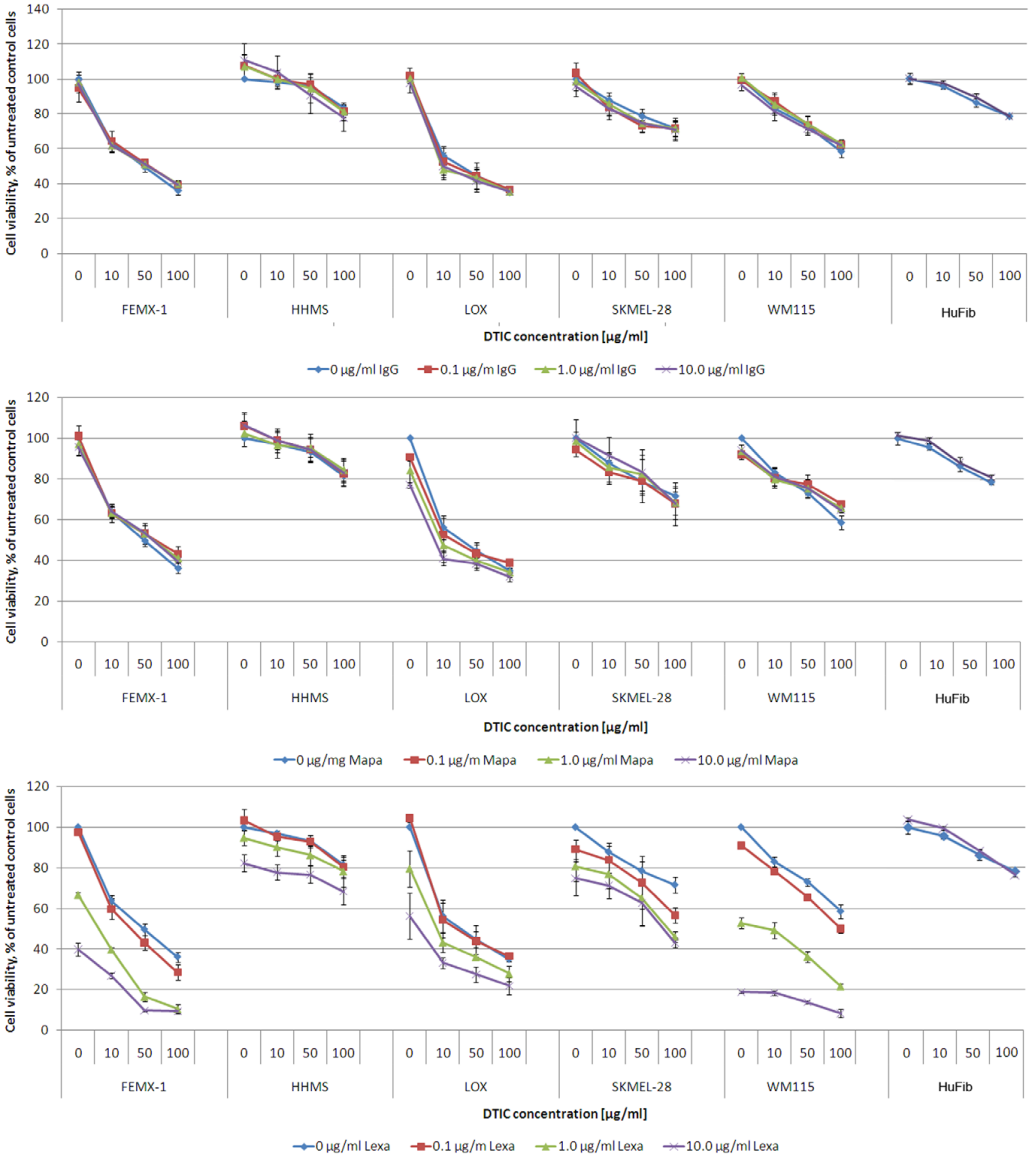
The sensitizing effect of agonistic TRAIL receptor antibodies on DTIC induced cell death. Five different melanoma cell lines were exposed to DTIC and/or indicated agonistic TRAIL receptor antibodies; A) isotype control; B) mapatumumab C) lexatumumab at designated concentrations, and the cell viability was measured after 72 h. The percentage of living cells is expressed relative to untreated control cells. Four parallels were included in each assay, and at least three separate biological experiments were performed. The data are presented as mean ± SEM.

DTIC has for many years been the standard drug for treatment of metastatic melanoma, but unfortunately the response rate is low [19]. Based on previously published data showing synergistic effect of combining AdhCMV-TRAIL and DTIC [20], our aim in this study was to explore the effect of the agonistic antibodies on DTIC sensitivity. We applied different concentrations of IgG isotype control, mapatumumab and lexatumumab, and combined with increasing concentrations of DTIC in selected melanoma cell lines (Figure 2). The effect of the combination was also evaluated in normal human fibroblast cells, HuFib. None of the antibodies induced additional cell death compared to what induced by the DTIC alone in HuFib, and similar results were observed for the IgG isotype control (Figure 2A) and mapatumumab (Figure 2B) in the melanoma cell lines.

However, in the melanoma cell lines exposed to lexatumumab and DTIC, a shift in the curve was noted compared to DTIC mono treatment (Figure 2C), indicating that the surviving fraction was reduced. The combinational effect was determined by calculation of the combination index (CI) value using Calcusyn software (Table S2). The CI values implied that DTIC and lexatumumab act synergistically, with a tendency to additive or antagonistic effects at the highest lexatumumab concentration in some cell lines.

### Reduced Clonogenicity After Combined Treatment with Lexatumumab and DTIC

**Figure 3:**
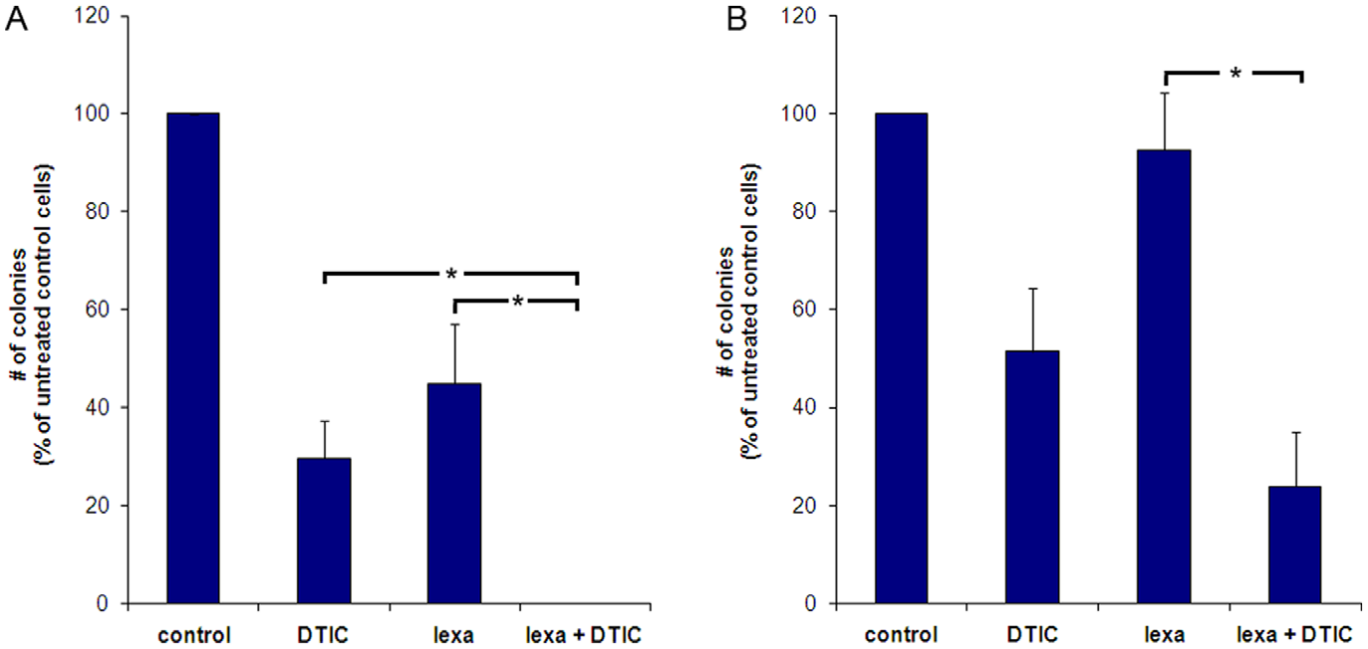
Clonogenic ability in vitro after lexatumumab (1 µg/ml) and DTIC (50 µg/ml) treatment of the cell lines. (A) FEMX-1 and (B) HHMS. The cells were seeded in six well plates and the next day exposed to the indicated treatment. Two weeks later the colonies were fixed, stained with 0.04% crystal violet and counted. Results are expressed relative to untreated control cells and are presented as mean ± standard error of the mean (SEM) of three parallel experiments. Significant difference in clonogenicity after combination treatments relative to DTIC or lexatumumab mono-treatment is indicated by (*).

Two cell lines, FEMX-1 and HHMS, with good and moderate response to the combination therapy, respectively, were selected for further studies to improve the understanding of the synergistic mechanism. Initially, the long term effect of the treatment was explored by clonogenic assay. Cells were seeded at low concentrations, exposed to the indicated treatments and colony formation was evaluated after two weeks (Figure 3). Comparable to the effect observed in the MTS assay, the combination treatment significantly reduced the colony formation compared to mono therapy in FEMX-1 (p = 0.04 DTIC and p = 0.02 lexatumumab), and a more modest response was observed in HHMS (p = 0.03 lexatumumab). Interestingly, the effect of both single agent and combination therapies were stronger in the clonogenic assay than observed in the MTS-assay in both cell lines.

### Combining Lexatumumab and DTIC Enhance the Level of Caspase Dependent Apoptosis in Sensitive Cells

**Figure 4:**
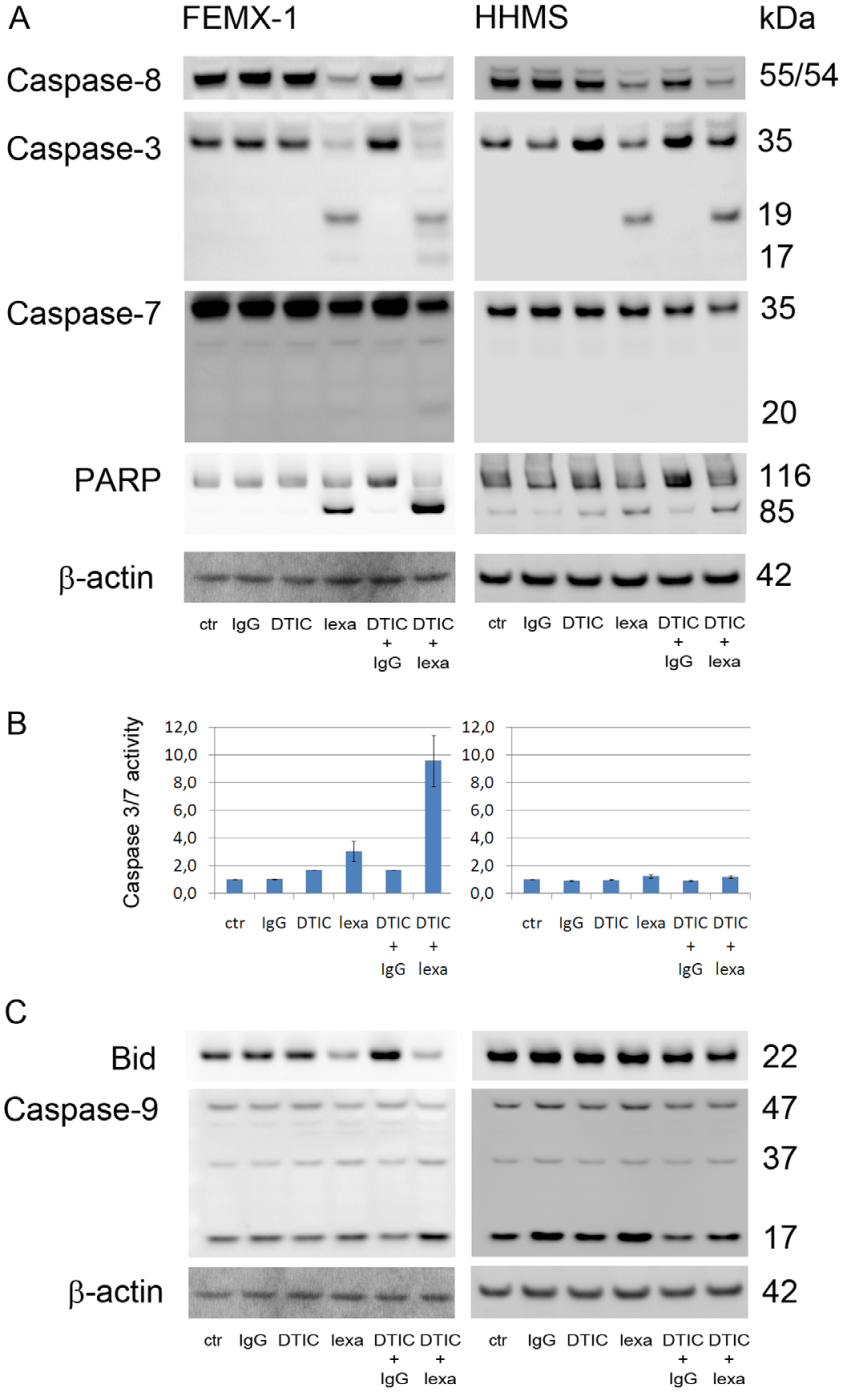
Evaluation of caspase activation after lexatumumab (1 µg/ml) and DTIC (50 µg/ml) treatment in FEMX-1 and HHMS. The cells were harvested 24 h (Western blot) or 48 h (Caspase activity) after treatment exposure and analyzed for various apoptotic markers. (A) Immuno-blot detection of caspase-8, −3 and −7 and PARP cleavage. (B) Caspase 3/7 activity. Results are expressed relative to untreated control cells and normalized to the cell number in the sample. Data are presented as mean ± standard error of the mean (SEM) of three parallel experiments. (C) Immuno-blot detection of Bid and caspase-9 cleavage. β-actin is used as loading control in (A) and (C). Cell lysates were prepared from three independent biological experiments, and representative blots are included in the figure.

Subsequently, the activation of initiator and effector caspases was investigated by Western blot. The extrinsic caspase cascade was initiated upon lexatumumab treatment in both FEMX-1 and HHMS cells indicated with cleavage of the initiator caspase-8 (Figure 4A). Treating the cells with both DTIC and lexatumumab induced a stronger processing of caspase-8 in the FEMX-1 cells, and also the appearance of bands representing cleavage products of caspase-3 and -7, corresponding to the active forms of the caspases. The activation of the effector caspases in the FEMX-1 cell line coincided with significant processing of PARP and a strong signal in the caspase 3/7 activity assay (Figure 4B). In the HHMS cells only weak cleavage of caspase-3 and −7 and PARP were observed and very limited caspase 3/7 activity were measured.

Furthermore, the role of the intrinsic pathway was studied by evaluation of Bid cleavage and activation of pro-caspase 9 (Figure 4C). Lexatumumab induced strong cleavage of Bid both alone and in combination with DTIC in the FEMX-1 cell line, while only a weak reduction in Bid level was observed after combination treatment in HHMS cells. Both FEMX-1 and HHMS expressed significant levels of the active forms of caspase-9 (37 kDa or 17 kDa) in the control samples, possibly due to autoactivation. An increase in the level of fully cleaved caspase-9 was detected after the combination treatment in FEMX-1, while expression comparable to the control cells was observed in HHMS cells, suggesting involvement of the intrinsic pathway only in the FEMX-1 cells.

### Effect of DTIC and Lexatumumab on Bcl-2 Protein Family Expression in FEMX-1 Cells

**Figure 5:**
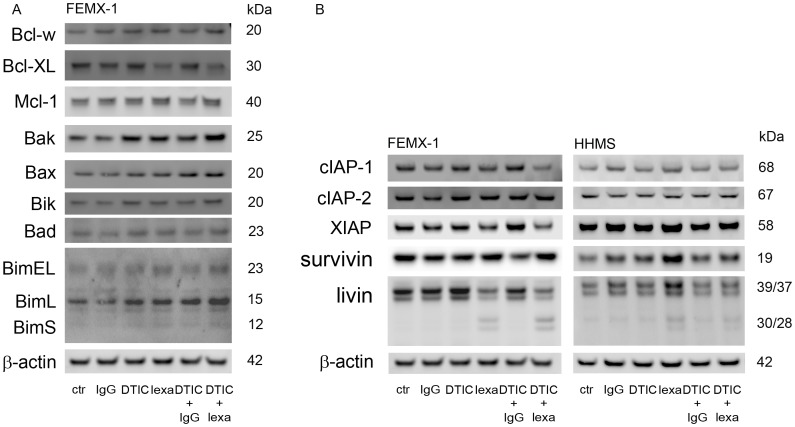
Evaluation of apoptosis regulating proteins after lexatumumab (1 µg/ml) and DTIC (50 µg/ml) treatment in FEMX-1 and HHMS. The cells were harvested 24 h after treatment exposure and analyzed for various apoptotic markers. (A) Immuno-blot detection of Bcl-w, Bcl-XL, Mcl-1, Bak, Bax, Bik, Bad, BimEL, BimL and BimS expression in FEMX-1 cells. (B) Immuno-blot detection of cIAP-1, cIAP-2, XIAP, survivin and livin in FEMX-1 and HHMS cells. β-actin is used as loading control. Cell lysates were prepared from three independent biological experiments, and representative blots are included in the figure.

As shown in Figure 4C the cleavage of Bid is comparable in FEMX-1 both for lexatumumab mono treatment and in combination with DTIC, but enhanced expression of fully cleaved caspase-9 is observed only after adding DTIC. The main regulators of the intrinsic pathway are members of the Bcl-2 family controlling the mitochondrial membrane integrity. The expression of selected family members was evaluated to detect any changes in expression as a result of the treatment (Figure 5A). The level of the anti-apoptotic proteins Bcl-w and Mcl-1 is not affected by the treatments, while Bcl-XL seems to be down-regulated in both lexatumumab and lexatumumab + DTIC treated samples. No differences in expression of the pro-apoptotic proteins BimEL, BimS, Bik and Bad can be seen in Figure 5A, while the levels of Bak, Bax and BimL were enhanced in samples treated with both lexatumumab and DTIC compared to control cells. In summary, the results suggest a shift in the balance between pro- and anti-apoptotic proteins in favor of enhanced activation of the intrinsic pathway.

### Effect of DTIC and Lexatumumab on Inhibitor of Apoptosis Proteins (IAPs) Expression

Another apoptosis regulating protein family is the Inhibitor of Apoptosis Proteins (IAPs), having the ability to affect both initiator and effector caspases. The expression of five different IAPs was studied in both cell lines (Figure 5B), and the expression was most influenced in the FEMX-1 cells. The expression of survivin and cIAP-2 was more or less unchanged while a reduction in the expression of cIAP-1 was observed only in the sample exposed to the combination treatment. XIAP and livin expression was reduced in the samples treated with lexatumumab alone or in combination with DTIC, and interestingly, a cleavage of livin into its pro-apoptotic form was observed. The expression of IAP members in the HHMS cells is in general unaffected by the different treatments, apart for a weak cleavage of livin in lexatumumab treated samples. Thus, shift in the expression of pro- and anti-apoptotic proteins may explain the enhanced induction of apoptosis in FEMX-1, and the lack of changes explains the low level of apoptosis in HHMS.

### DTIC Demonstrated Increased Antitumor Effect *in vivo* when Combined with Lexatumumab

**Figure 6:**
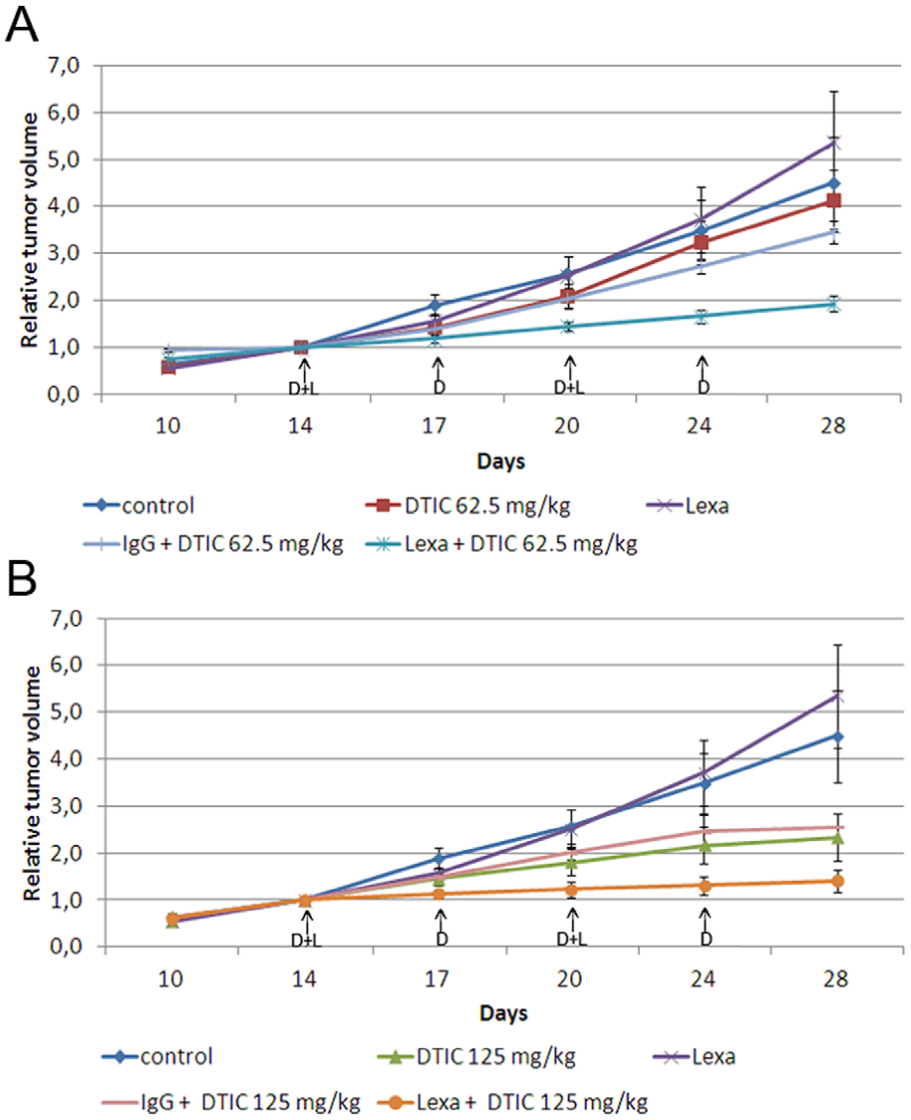
The growth reducing capacity of the combination treatment in vivo. FEMX-1 xenografts were treated with lexatumumab (L) (10 mg/kg, twice a week, i.v. injection), IgG isotype control (10 mg/kg, twice a week, i.v. injection), DTIC (D) ((A) 62.5 mg/kg or (B) 125 mg/kg once a week, i.p. injection) or the combination of the Abs and DTIC. Treatment time is indicated in the figure by arrows. The tumor volumes were measured twice a week using a caliper, and are presented as relative tumor volume related to the volume of the tumor at the initiation of the treatment. At least eight tumors are included in each treatment group.

Our *in vitro* data demonstrated that DTIC in combination with lexatumumab increased the toxic effect. To determine whether this effect could be reproduced *in vivo* the growth of FEMX-1 xenografts was followed while treating the mice with either lexatumumab (10 mg/kg, i.v.) twice a week, two different concentrations of DTIC (either 62.5 or 125 mg/kg, i.p.) once a week or the combination of lexatumumab and DTIC or IgG isotype control and DTIC (Figure 6). The number of mice included in each treatment group is reported in Table S3. Lexatumumab treated xenografts showed comparable growth as the control tumors, while DTIC treated tumors showed a dose dependent growth reduction. Mice treated with DTIC in combination with the IgG isotype control showed similar growth curves as DTIC mono therapy. Combining lexatumumab and DTIC induced a significant reduction in the tumor growth compared to the growth of the tumors treated with DTIC and IgG at the lowest DTIC concentration (Repeated measures, Dunnet's test for adjustment for multiple comparisons, p = 0.003), while a tendency to reduced growth was observed for the highest DTIC dose (p = 0.064). However, the growth of the three other groups (control, lexatumumab and DTIC) was not significantly different from the growth of lexatumumab and DTIC due to high variance in these groups. To increase the statistical test power the control groups were combined; group 1: untreated tumors and lexatumumab treated tumors, groupt 2: DTIC-treated and DTIC- and IgG-treated tumors. Comparing the growth of these merged control groups with the growth of the tumors treated with lexatumumab and DTIC a significant reduction in tumor growth was observed relative to both control groups and for both DTIC concentrations (Repeated measures, Dunnet’s test for adjustment for multiple comparisons, DTIC 62.5 mg/kg: group 1: p = 0.02, group 2: p = 0.02. DTIC 125 mg/kg: group1: p = 0.000, group 2: p = 0.035).

When evaluating the growth curves, none of the treatments seemed to reduce the tumor volume, suggesting a cytostatic rather than a cytotoxic effect. However, many of the tumors regressed to various degrees below the initial volume they possessed at the treatment initiation (data not shown). The regression tended to be temporarily, but indicate that the combination can have a cytotoxic effect.

### Effect of DTIC and Lexatumumab on the Expression on Apoptosis Relevant Proteins

**Figure 7:**
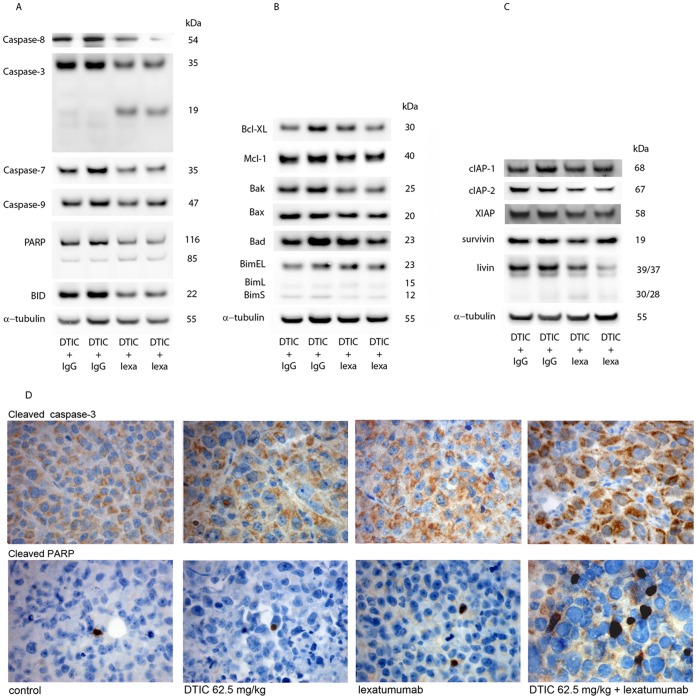
Expression of caspases and apoptosis regulating proteins after treating xenografts with lexatumumab (10 mg/kg, twice a week, i.v. injection) and DTIC (62.5 mg/kgonce a week, i.p. injection) or with IgG isotype control (10 mg/kg, twice a week, i.v. injection) and DTIC (62.5 mg/kg once a week, i.p. injection. The FEMX-1 xenografts were harvested and cell lysates were prepared for Western blot or for IHC. (A) Immuno-blot detection of caspase-8, −3, −7 and −9 and Bid and PARP cleavage. (B) Immuno-blot detection of Bcl-XL, Mcl-1, Bak, Bax, Bad, BimEL, BimL and BimS. (C) Immuno-blot detection of cIAP-1, cIAP-2, XIAP, survivin and livin. α-tubulin is used as loading control. Cell lysates were prepared from four independent xenorgafts. (D) Immunohistochemical detection of cleaved caspase-3 and PARP in FEMX-1 xenografts treated as indicated in the figure.

To study the molecular effect of the combination treatment *in vivo, c*ell lysates were prepared from xenografts treated with DTIC and IgG and with DTIC and lexatumumab. Figure 7A show that DTIC in combination with lexatumumab cleaved pro-caspase-3, −7, −8 and −9, while very little cleavage was observed in the xenografts receiving DTIC and IgG. The increased caspase activity was accompanied with enhanced Bid and PARP cleavage. The expression of various Bcl-2 family members was comparable between the two groups (Figure 7B), while the IAP expression show different expression pattern (Figure 7C). Both cIAP-2 and livin, were down regulated in the xenografts treated with DTIC and lexatumumab. The expression of cleaved caspase-3 and cleaved PARP was also monitored by immunohistochemistry (IHC) (Figure 7D). Whereas only a few cleaved PARP positive cells were detected in lexatumumab and DTIC alone treated tumors, a clear increase in percentage of immunoreactive cells were seen in tumors having received combination treatment. Cleaved caspase-3 was on the other hand detected in the majority of both controls and treated tumor, although an increase in staining intensity was observed following combinational treatment.

## Discussion

Metastatic melanoma cells appear usually resistant to chemo- and radiotherapy. Despite new promising drugs emerging, as Ipilimumab and various BRAF inhibitors, the need for alternative treatment options is immense. One promising new anticancer drug is agonistic TRAIL receptor antibodies and several agonists are currently being evaluated in early phase clinical trials in a variety of cancer types. Stable disease and partial responses have been reported after administration of mapatumumab or lexatumumab as mono therapy or in combination with chemotherapy in phase I and II trials [21], [22], [23], [24], [25], [26], and both antibodies were well tolerated. The effect of these antibodies have, however, not previously been studied in melanoma. The present evaluation in a panel of melanoma cell lines showed that mapatumumab had low or no impact on the cell viability, and that lack of effect could be related to low expression of the receptor DR4. Low DR4 expression level is in agreement with previous reports examining the expression both in melanoma cell lines and patient materials [8]. LOX was the only one of our cell lines that expressed DR4 and responded to mapatumumab treatment. In contrast, the DR5 targeted antibody lexatumumab induced a variable, but dose-dependent reduction in cell viability in all cell lines studied. Several mechanisms causing resistance for TRAIL-induced apoptosis have been reported, including low expression of DR4, DR5 or the initiator caspase-8, high expression of anti-apoptotic molecules as c-FLIP [8], IAPs or BcL-2 family members, incomplete processing of caspase-3 [27], [28], [29], activation of NFκΒ mediated survival mechanisms [30], [31] and loss of pro-apoptotic proteins. In addition, lack of cross-linking to the cell membrane may reduce the apoptosis inducing activity of TRAIL [32]. In an attempt to enhance the antitumor efficacy, combination therapy with the standard chemotherapy for melanoma patients, DTIC, was investigated. Combination treatment that increases the percentage of DTIC-responder or the progression free survival would be of high clinical value. We have previously obtained a synergistic effect by combining AdhCMV-TRAIL and DTIC [20], and Ding *et al*. showed recently an enhanced anticancer activity *in vitro* when delivering anti-DR5 antibodies covalently linked to DTIC-loaded nanoparticles [33]. No benefit from combining mapatumumab with DTIC was observed, implying that DTIC did not influence the sensitivity of cells to mapatumumab treatment, e.g. by altering the expression of DR4 on the cell surface. This is in line with our earlier results showing that DTIC does not affect the membrane expression of DR4 (or DR5) [20]. In contrast, combining DTIC and lexatumumab induced synergistic effects on cell viability and clonogenicity *in vitro*. Noticeably, the studied combination did not induce additional toxicity to normal human fibroblast, HuFib, than DTIC treatment alone.

To observe whether the effects from *in vitro* cultures could be confirmed *in vivo*, FEMX-1 xenografts were used. The growth of the tumors treated with both DTIC and lexatumumab was significantly reduced compared to the combined control group consisting of untreated tumors and lexatumumab treated tumors. And more importantly, as the main question of this paper is if the addition of lexatumumab will improve the effect of DTIC, a significant reduction for both DTIC concentrations was observed when comparing the growth with the merged group of tumors treated either with DTIC alone or DTIC and the control antibody IgG. These results suggest that lexatumumab can have the potential to increase the efficacy of DTIC in the clinic.

TRAIL sensitivity has been related to sufficient processing of caspase-3 [27], [28], [29]. The activation of caspase-3 is initiated by caspase-8 leading to a 19 kDa enzymatically inactive intermediate form. Subsequently, the p19 is processed, most likely due to autocatalysis, into active caspase-3 [34]. This activation cascade was clearly visible in the FEMX-1 cells after lexatumumab exposure *in vitro*, and even more pronounced after the combination therapy, while neither the active caspase-3 band nor caspase-3 activity were present in the less sensitive HHMS cells. However, high levels of the 19 kDa intermediate product and efficient caspase-8 processing were observed. Thus, the reduced sensitivity to lexatumumab and DTIC in HHMS could possibly be accounted for by a blockage in the caspase-3 autocatalytic activity.

XIAP is known to directly interact with caspase-3 and -7, preventing full processing into the active form [35]. The expression of XIAP in the HHMS cells is not affected by the different treatments, while a reduced level is observed in FEMX-1 cells treated with lexatumumab alone or in combination with DTIC. In addition, livin was down-regulated. Livin has been related to Smac sequestration, reducing the levels of Smac available for binding to XIAP [36], [37]. Thereby, depletion of available XIAP liberates caspase-3 and -7 to be processed into its active residues as observed in the FEMX-1 cell line, but not in HHMS. We have recently shown that siRNA mediated down regulation of XIAP sensitizes melanoma cells to TRAIL induced cell death, implying an important role of XIAP [38]. Furthermore, Hörnle et al. recently showed that activated caspase-3 cleaves XIAP and further enhances the activation of caspase-3 in a positive feedback loop [28]. This mechanism was found involved both in cisplatin- and UVB- induced sensitization to TRAIL in melanoma cells, and it is worth speculating whether a similar mechanism may be responsible for the observed synergy between DTIC and TRAIL. Increased cleaved caspase-3 expression was also observed in vivo both by Western analysis and IHC.

Additional changes in IAP expression were observed in the FEMX-1 cells. Administration of lexatumumab induced cleavage of livin into its truncated, proapoptotic form, a compound previously shown to accelerate apoptosis [39], [40]. The cleavage was observed clearly *in vitro* and only modestly *in vivo*. Additionally, down regulation of cIAP-1 was observed in samples exposed to the combined treatment *in vitro*, while cIAP-2 expression was reduced *in vivo*. Both cIAP-1 and cIAP-2 are involved in ubiquitin-dependent signaling events that regulate NF*κ*B and is required for activation of the canonical pathway as well as suppression of the non-canonical NF*κ*B pathway. Degradation of these IAPs has been related to stabilization of the NF*κ*B inducing kinase (NIK), thereby causing spontaneous activation of the non-canonical NF*κ*B pathway and subsequent production of autocrine TNF*α* followed by induction of cell death [41], [42].

In addition to reduced levels of IAPs, the combination treatment induced increased activation of the intrinsic apoptotic pathway in FEMX-1, demonstrated by enhanced caspase-9 processing. The main regulators of the intrinsic pathway are members of the BcL2-family, and tBid and Bim are key signaling intermediates [43], [44]. They trigger mitochondrial outer membrane permeabilization by promoting oligomerization of the pro-apoptotic Bak and/or Bax within this membrane. Enhanced cleavage of Bid was observed and Bak, Bax and BimL were all up regulated upon the combination treatment. Up regulation of Bax has been reported as an important player for the enhanced efficacy of different combination treatments involving TRAIL, (e.g. radiation and chemotherapy [45], [46]). Furthermore, the combination resulted in down regulation of Bcl-XL. *In vivo* only cleavage of Bid was observed, while the expression of the other Bcl-2 family members was comparable to the xenografts treated with DTIC and IgG.

Altogether, the data presented suggest that a change in the balance between anti- and pro-apoptotic proteins drives the synergistic effect of DTIC-and TRAIL-induced cell death in the FEMX-1 cells, both *in vitro* and *in vivo*. The combination induced, however, only an additive effect in the most therapy resistant HHMS cells. In this cell line limited activation of executor caspases was observed, thus targeting XIAP in combination with lexatumumab and DTIC might possibly enhance the treatment effect.

In summary, the data presented herein demonstrates that the combination of DTIC and lexatumumab induced additive or synergistic effects on cell death in all the melanoma cell lines tested. The combination regimen represents a possible strategy to increase DTIC sensitivity in melanoma cells, and could potentially be helpful for a large group of patients having no curative treatment protocols at present. Further elucidation of the mechanism involved and evaluation of the efficacy of DTIC and lexatumumab treatment in other *in vivo* models are warranted to investigate its applicability and potential for clinical implication.

## Supporting Information

Table S1 **Expression of DR4 and DR5 in five selected melanoma cell lines analyzed by flow cytometry.** Fluorescence intensity is presented relative to control. Control samples were incubated with the secondary Ab. At least three separate biological experiments were performed, and the data are presented as mean ± SEM.
(DOCX)

Table S2 **The combination index (CI) value calculated using Calcusyn software after DTIC and lexatumumab treatment of selected melanoma cell lines.** Synergistic values in bold.
(DOCX)

Table S3 **The number of tumors and mice included in each treatment group.** Tumor cells were injected s.c. on each flank of the mouse, and generally two tumors were established on each mouse. For the mice with two tumors, the mean value was used for statistical analysis.
(DOCX)
