# Supplementary material for: Dacarbazine and the Agonistic TRAIL Receptor-2 Antibody Lexatumumab Induce Synergistic Anticancer Effects in Melanoma
Source: PLoS One. 2012 Sep 20;7(9):e45492. doi: 10.1371/journal.pone.0045492 (PMC3447808; doi:10.1371/journal.pone.0045492)
Supplement: Table S1 — Expression of DR4 and DR5 in five selected melanoma cell lines analyzed by flow cytometry. Fluorescence intensity is presented relative to control. Control samples were incubated with the secondary Ab. At least three separate biological experiments were performed, and the data are presented as mean ± SEM. (DOCX) [file pone.0045492.s001.docx]

*Supplementary table 1: Expression of DR4 and DR5 in five selected melanoma cell lines analyzed by flow cytometry. Fluorescence intensity is presented relative to control. Control samples were incubated with the secondary Ab. At least three separate biological experiments were performed, and the data are presented as mean ± SEM.*

| **Cells** | **Control** | **DR4** | **DR5** |
| --- | --- | --- | --- |
| **FEMX-1** | 1.0 | 1.3±0.3 | 9.6±1.0 |
| **HHMS** | 1.0 | 1.1±0.1 | 7.8±1.2 |
| **LOX** | 1.0 | 13.1±1.4 | 15.4±1.0 |
| **SKMEL-28** | 1.0 | 1.1±0.1 | 3.9±0.6 |
| **WM115** | 1.0 | 0.8±0.1 | 19.8±3.0 |
