# Supplementary material for: Dacarbazine and the Agonistic TRAIL Receptor-2 Antibody Lexatumumab Induce Synergistic Anticancer Effects in Melanoma
Source: PLoS One. 2012 Sep 20;7(9):e45492. doi: 10.1371/journal.pone.0045492 (PMC3447808; doi:10.1371/journal.pone.0045492)
Supplement: Table S2 — The combination index (CI) value calculated using Calcusyn software after DTIC and lexatumumab treatment of selected melanoma cell lines. Synergistic values in bold. (DOCX) [file pone.0045492.s002.docx]

*Supplementary table 2: The combination index (CI) value calculated using Calcusyn software after DTIC and lexatumumab treatment of selected melanoma cell lines. Synergistic values in bold.*

|  | | **CI values** | | | | |
| --- | --- | --- | --- | --- | --- | --- |
| **DTIC [µg/ml]** | **Lexatumumab [µg/ml]** | **FEMX-1** | **HHMS** | **LOX** | **SKMEL-28** | **WM115** |
| 10 | 0,1 | **0,8** | **0,4** | **0,6** | **0,4** | 1,2 |
| 10 | 1 | **0,3** | **0,4** | **0,5** | **0,4** | **0,8** |
| 10 | 10 | **0,8** | 2,2 | 2,1 | **0,7** | 1,1 |
| 50 | 0,1 | 1,1 | 1,2 | 1,2 | **0,6** | 1,5 |
| 50 | 1 | **0,1** | 1,0 | **0,9** | **0,5** | **0,6** |
| 50 | 10 | **0,2** | 3,1 | 2,6 | **0,5** | **0,8** |
| 100 | 0,1 | **0,6** | **0,9** | 1,3 | **0,6** | 1,0 |
| 100 | 1 | **0,1** | 1,1 | **0,9** | **0,4** | **0,3** |
| 100 | 10 | **0,2** | 3,4 | 2,6 | **0,4** | **0,4** |
