# Supplementary material for: Dacarbazine and the Agonistic TRAIL Receptor-2 Antibody Lexatumumab Induce Synergistic Anticancer Effects in Melanoma
Source: PLoS One. 2012 Sep 20;7(9):e45492. doi: 10.1371/journal.pone.0045492 (PMC3447808; doi:10.1371/journal.pone.0045492)
Supplement: Table S3 — The number of tumors and mice included in each treatment group. Tumor cells were injected s.c. on each flank of the mouse, and generally two tumors were established on each mouse. For the mice with two tumors, the mean value was used for statistical analysis. (DOCX) [file pone.0045492.s003.docx]

*Supplementary table 3: The number of tumors and mice included in each treatment group. Tumor cells were injected s.c. on each flank of the mouse, and generally two tumors were established on each mouse. For the mice with two tumors, the mean value was used for statistical analysis.*

| **Treatment group** | **# tumors** | **# mice** |
| --- | --- | --- |
| Control | 19 | 10 |
| DTIC 62.5 mg/kg | 19 | 10 |
| DTIC 125 mg/kg | 16 | 10 |
| Lexatumumab | 21 | 12 |
| IgG + DTIC 62.5 mg/kg | 10 | 5 |
| IgG + DTIC 125 mg/kg | 8 | 4 |
| Lexatumumab + DTIC 62.5 mg/kg | 29 | 15 |
| Lexatumumab + DTIC 125 mg/kg | 20 | 11 |
